# Enteric Methane Emissions of Dairy Cows Predicted from Fatty Acid Profiles of Milk, Cream, Cheese, Ricotta, Whey, and Scotta

**DOI:** 10.3390/ani10010061

**Published:** 2019-12-29

**Authors:** Giovanni Bittante, Matteo Bergamaschi

**Affiliations:** Department of Agronomy, Food, Natural Resources, Animals and Environment (DAFNAE), University of Padova, Viale dell’Università 16, 35020 Padova, Italy; mbergam@ncsu.edu

**Keywords:** ecological footprint, greenhouse gases, global warming, cheese environmental impact, dairy products

## Abstract

**Simple Summary:**

Six main fatty acids measured by gas-chromatography of four types of milk samples, fresh products, by-products, and ripened cheeses were used for predicting enteric methane yield per kg of feed consumed and intensity per kg of milk produced. Methane yield and intensity can be predicted from single milk samples with good accuracy. Cream, ricotta, and ripened cheese could be used only taking into account the possible overestimation of emissions and increasing the number of samples analyzed to improve the precision. Among by-products, whey could be a possible alternative source of information for predicting methane emission, whereas scotta showed low precision. Ripened cheeses were found to be less valuable sources of information to predict methane emission. This method could be used for monitoring the ecological footprint of different farms, dairy and feeding systems, and processing units.

**Abstract:**

Enteric methane emissions (EME) of ruminants contribute to global climate change, but any attempt to reduce it will need an easy, inexpensive, and accurate method of quantification. We used a promising indirect method for estimating EMEs of lactating dairy cows based on the analysis of the fatty acid (FA) profile of their milk. The aim of this preliminary study was to assess milk from four single samplings (morning whole, evening whole, evening partially skimmed, and vat milks) as alternatives to reference whole milk samples from two milkings. Three fresh products (cream, cheese, and ricotta), two by-products (whey and scotta), and two long-ripened cheeses (6 and 12 months) were also assessed as alternative sources of information to reference milk. The 11 alternative matrices were obtained from seven experimental cheese- and ricotta-making sessions carried out every two weeks following the artisanal Malga cheese-making procedure using milk from 148 dairy cows kept on summer highland pastures. A total of 131 samples of milk, dairy products, and by-products were analyzed to determine the milk composition and to obtain detailed FA profiles using bi-dimensional gas-chromatography. Two equations taken from a published meta-analysis of methane emissions measured in the respiration chambers of cows on 30 different diets were applied to the proportions of butyric, iso-palmitic, iso-oleic, vaccenic, oleic, and linoleic acids out of total FAs to predict methane yield per kg of dry matter ingested and methane intensity per kg of fat and protein corrected milk produced by the cows. Methane yield and intensity could be predicted from single milk samples with good accuracy (trueness and precision) with respect to those predicted from reference milk. The fresh products (cream, cheese and ricotta) generally showed good levels of trueness but low precision for predicting both EME traits, which means that a greater number of samples needs to be analyzed. Among by-products, whey could be a viable alternative source of information for predicting both EME traits, whereas scotta overestimated both traits and showed low precision (due also to its very low fat content). Long-ripened cheeses were found to be less valuable sources of information, although six-month cheese could, with specific correction factors, be acceptable sources of information for predicting the methane yield of lactating cows. These preliminary results need to be confirmed by further study on different dairy systems and cheese-making technologies but offer new insight into a possible easy method for monitoring the EME at the field level along the dairy chain.

## 1. Introduction

Enteric methane (EME) emitted by lactating cows [1] makes the greatest contribution to greenhouse gas (GHG) emissions out of the entire animal sector, and therefore has the greatest impact on climate change [2].

The direct quantification of GHG using respiration chambers requires facilities, tools, resources, and knowledge that are available in only a few research centers [3] and make it impossible to test GHG emissions in the field with a large number of farms and cows. Among the different proxies that have been proposed for indirectly measuring EME in dairy cattle [4], the analysis of fatty acid (FA) profiles of milk and a proper combination of FAs constitute an easy-to-use method for use in the field [5,6,7], as it requires only the collection of milk samples and analysis of them in the laboratory [8]. This method exploits the complex relationships between feed characteristics, rumen microbial activity, methane production, the production and metabolism of volatile and non-volatile fatty acids, and their absorption and transportation to the udder, de novo synthesis of fatty acids in the mammary gland, and, lastly, fat globule excretion in milk [5,9,10].

Van Lingen et al. [11] undertook a meta-analysis of the relationships between EME and milk FA profiles using the combined data from eight experiments covering 30 different diets, from which they derived two equations, one for predicting methane yield per kg of dry matter (DM) intake (CH_4_/DMI) and one for methane intensity (CH_4_/CM) per kg of fat-protein corrected milk (CM). In a previous study [12], we applied these equations to data from a large survey to analyze the effects of dairy system, herd within dairy system and individual cow factors (parity and DIM) on methane production (g/d), methane yield (g/kg DMI), and methane intensity per kg of corrected milk, of fresh cheese and of cheese solids contained in fresh cheese. A similar meta-analysis was carried out recently [13] for predicting also daily methane production (dCH_4_) per cow.

Although an approach based on sampling milk from individual cows is of interest with regard to possible genetic improvement of dairy cattle aimed at GHG mitigation [7,13,14], bulk samples collected during milk processing could also be useful for easily monitoring different suppliers. Moreover, as the prediction equations are based on the proportions of specific fatty acids (FAs) out of the total amount of FAs in untreated whole milk representative of daily production, we hypothesize that these equations could also be applied to the FA profiles of other milk samples more easily collected, and of cheese-making products and by-products with very different fat contents, provided the proportions of the different fatty acids are very similar to those of the milk used for processing. This would allow EMEs to be easily monitored across the entire dairy chain. In this first preliminary study, we aimed to compare the EME estimates obtained from reference daily milk (the average of whole unprocessed milk obtained from the morning and evening milkings), with those from four types of milk from single samplings, and seven dairy products and by-products obtained from a series of artisanal cheese- and ricotta-makings. The specific aims were: (i) to compare single milk samples obtained during milk processing vs. reference milk for estimating methane yield (CH_4_/DMI, g/kg) and methane intensity (CH_4_/CM, g/kg); (ii) to assess the accuracy of predicting EME traits directly from the fatty acid profiles of the products and by-products of cheese- and ricotta-making vs. reference milk.

## 2. Material and Methods

### 2.1. Farms and Animals

This preliminary study is part of the Juribello project on artisanal production of Malga cheese from the milk of cows grazing on summer highland pastures in the Alps. Details of the project and of the farm and pasture are given in a previous study [15]. Briefly, the study was carried out from June to September during summer highland grazing (Malga Juribello, Paneveggio-Pale di San Martino Nature Park, Trento, Italy) on a temporary summer farm equipped for processing milk to produce traditional “Malga” (summer Alpine pasture) cheese. The milk was obtained from 148 cows of different breeds (Brown Swiss, Holstein Friesian, Simmental, Rendena) from 12 permanent lowland farms. Production of the cows at the beginning of pasture was 23.6 ± 5.7 kg/day. All the cows were in mid- and late-lactation (days in milk: 233 ± 90 day) and various parities (2.4 ± 1.7). The cows were milked twice daily at 12-hour interval and managed according to farm practice and no specific intervention on animals was done.

### 2.2. Cheese and Ricotta Making

A total of seven cheese- and ricotta-making sessions were carried out using bulk milk collected every two weeks during summer pasture. The procedure used has been described in detail in a previous paper [15]. Briefly, according to the procedure used in the study area for producing local typical Malga cheese (a semi-skimmed hard cheese), 250 L of raw whole milk from the evening milking was put in an open flat tank for overnight natural creaming. The following morning, the surface layer of cream was skimmed from the milk, which was then transferred to a vat, mixed with 250 L of freshly milked morning whole milk, heated, and supplemented with commercial rennet. The resulting curd was cut, turned to facilitate draining, cooked at 45 °C and then put into 8–9 molds (30 cm diameter × 12 cm high), pressed, salted in brine for 80 h, and ripened in a cellar for 6 or 12 mo. The whey was transferred to a smaller vat and heated to 90 °C before 0.750 L of vinegar was added to catalyze coagulation. The resulting traditional ricotta was separated, weighed, and placed into molds (20 cm diameter × 15 cm high) to drain off the scotta [15]. The product yield traits of cream, ricotta, and fresh and ripened cheese are summarized in Table 1.

### 2.3. Sampling

This research is based on data and samples collected from a commercial farm without animal manipulation other than usual management (feeding, milking) of dairy cows and do not imply ethical concerns. A total of 11 milks and dairy products were sampled from each of the seven cheese-making sessions [16]: four types of raw milk (evening whole milk, evening skimmed milk, morning whole milk, and vat milk that is a mixture of evening skimmed and morning whole milk); three fresh products (cream, fresh cheese, ricotta); two liquid by-products (whey, scotta); and two ripened cheeses (6 and 12 months). A total of 133 samples were stored at −20 °C until analysis: one sample was collected for every cheese-making session for each one of the four types of milk, two for each other dairy products and whey, and three for the scotta, because of its very low fat content.

### 2.4. Quality Traits and Fatty Acid Profiles

The chemical traits of the milk, cream, whey, ricotta, scotta, curd and cheese were analyzed at Department of Agronomy, Food, Natural resources, Animals and Environment (DAFNAE), University of Padova (Legnaro, Padova, Italy). The fat, protein, casein, and lactose percentages of the milk samples were determined using a MilkoScan FT2 (Foss Electric A/S, Hillerød, Denmark) (Table 1), while the somatic cell content (SCC) was estimated using a Fossomatic FC automatic cell counter (Foss Electric A/S, Hillerød, Denmark) and then converted to somatic cell score (SCS) by logarithmic transformation [SCS = log_2_ (SCC/10,000) + 3].

### 2.5. Lipid Extraction and Esterification

Details of the fatty acid profiling of the 11 dairy matrices are given in a previous study [17]. Briefly, lipids were extracted from the dairy products according to [18,19] using hexane: isopropanol (3:2, vol/vol) as the solvent solution at room temperature. After evaporation of the solvent, the resulting extracted fat material was weighed. Lipids were extracted from the cream, curd, ricotta, and cheese using a Soxtec extraction apparatus (ST 255; Foss Electric) according to [20] methodology. All samples were transesterified and methylated according to [21].

### 2.6. GC × GC Analysis

Detailed fatty acid profiles were determined using GC × GC (7890A, Agilent Technologies, Santa Clara, CA, USA) with two columns in series and fitted with a modulator (G3486A CFT, Agilent Technologies, Santa Clara, CA, USA), and equipped with an automatic sampler (7693, Agilent Technologies, Santa Clara, CA, USA) and a flame ionization detector connected to the chromatography data system software (Agilent Chem Station, Agilent Technologies). Operating conditions, oven temperature program, valve and flame-ionization detector, and gas flow have been reported in detail by [22,23]. The resulting two-dimensional chromatograms were analyzed, and the peak cone volume of each FA calculated using the comprehensive GC × GC software (GC Image Software, Zoex Corp., Houston, TX, USA).

### 2.7. Identification and Quantification of FAs

The six FAs involved in EME traits estimations (butyric acid C4:0; iso-palmitic acid C16:0iso; iso-oleic acid C18:1*trans*10; vaccenic acid C18:1*trans*11; oleic acid C18:1*cis*9; linoleic acid C18:2*cis*9, *cis*12) were identified by comparing the peak cone positions in the chromatogram with those obtained from their GC reference standards [17]. Each FA was quantified in terms of the cone volume of each FA as a percentage of the volume of all FAs.

### 2.8. Estimation of Enteric Methane Emissions

The EMEs were estimated according to the equations derived by [11] from their meta-analysis. Equations recently developed by the same research group [13] were not used because they include some FAs not available in our dataset, or available but out of the range used for developing those equations. So, it was also not possible to compare the two sets of equations.

The equations used were:

- Methane yield, CH_4_/DMI, i.e., emission, in g per kg of DM intake, estimated according to the equation:
  CH_4_/DMI (g/kg) = 23.39 + 9.74 × C16:0*iso* − 1.06 × C18:1*t*10 + *t*11 − 1.75 × C18:2*c*9, *c*12
  where C16:0*iso* is iso-palmitic acid, C18:1*t*10 + *t*11 is the sum of iso-oleic and vaccenic acids, and C18:2*c*9, *c*12 is the linoleic acid content of milk, all expressed as % of the sum of all milk FAs.
- Methane intensity, CH_4_/CM, i.e., emission per unit of fat- and protein-corrected milk (CM), estimated according to the equation:
  CH_4_/CM (g/kg) = 21.13 − 1.38 × C4:0 + 8.53 × C16:0*iso* − 0.22 × C18:1*c*9 − 0.59 × C18:1*t*10 + *t*11
  where C4:0 is butyric acid, and C18:1c9 is oleic acid, both expressed as % of the sum of all milk FAs.

### 2.9. Statistical Analysis

The proportions of informative FAs (g/100 g FA), and the EME traits of the eleven dairy products collected from each of the seven cheese-making sessions and of the reference milk composition, obtained by averaging the values from the two daily milkings (whole evening and morning milk samples), were analyzed using SAS PROC MIXED (SAS Inst. Inc., Cary, NC, USA) and the following statistical model:

Y_ijk_ = μ + DP_i_ + date_j_ + e_ijk_

where Y_ijk_ is the individual FA content or EME trait; μ is the overall mean; DP_i_ is the fixed effect of the ith dairy product (i = 1 to 12); date_j_ is the repeated effect of the jth cheese-making session (j = 1 to 7); e_ijk_ is the residual random error term −N (0, σ^2^).

### 2.10. Definition of Accuracy, Trueness and Precision

We based our evaluation of alternative sampling methods on the following ISO definitions (ISO 5725-1:1994):

- accuracy measures the closeness of agreement between a test result and the accepted reference value and involves a combination of random components and a common systematic error or bias component;
- trueness measures the closeness of agreement between the average value obtained from a large series of test results and an accepted reference value (bias);
- precision measures the closeness of agreement between independent test results obtained under stipulated conditions.

So, the accuracy of the alternative samplings was defined in terms of trueness and precision.

To evaluate the trueness of the alternative sources of information, we calculated 11 contrasts between the reference value (average of the whole milk samples from the evening and morning milkings) and each of the 11 milks and dairy products and by-products sampled and analyzed. An alternative information source (single milk samples, fresh products, by-products and ripened cheeses) was assumed to have a high trueness for predicting EME traits if its least square mean (LSM) did not differ (*p* > 0.05) from the EME value of reference untreated whole daily milk.

We also calculated Pearson correlations between the methane yields and intensities of the reference milk samples and each of the 11 alternative matrices sampled. The precision of the alternative source of information was assumed to be:

- high if the correlation with the reference values was >80%;
- moderate if the correlation was 50% to 80%;
- low if the correlation was 20% to 50%;
- very low if the correlation was <20%.

## 3. Results

Table 2 shows the results of ANOVA analyzing the effects of source of information (reference milk, single milk samples, samples of dairy products and by-products) and cheese-making date on informative FAs and EME traits. 

The more important and variable FA was oleic acid, which in nine of the 11 matrices sampled differed significantly from the reference milk: Whole and partly evening skimmed milks had greater proportions of oleic acid, whereas all the other matrices, except vat milk and whey, had smaller contents. The differences in the proportions of oleic acid in the different dairy matrices tested did not, in any case, affect much the estimates of EME traits because oleic acid was not included in the equation predicting methane yield, and it had the lowest coefficient in the equation for estimating methane intensity per kg of corrected milk [11].

In contrast, iso-palmitic acid (C16:0iso) was the most predictive fatty acid, as it was the only one positively correlated with EME and had the greatest coefficient in both equations. In this case, the differences among the matrices analyzed were modest, and only cream and scotta contained a greater proportion of this FA than the reference milk.

The three fresh products (cream, ricotta and fresh cheese) and scotta, but not the ripened cheeses, had slightly greater contents of butyric acid than the reference milk. In contrast, the summed contents of iso-oleic and vaccenic acids were smaller in cream, scotta, and also ripened cheeses than in the reference milk. Lastly, linoleic acid content never differed from that in the reference milk.

As a result of the small and different- and sometimes opposite-changes in the proportions of the six informative FAs, and the compensations among them, there were no differences (high trueness) between the estimates of both EME traits obtained from the reference milk and those obtained not only from the four single milk samples (evening whole, evening partially skimmed, morning whole, and vat mix), but also from the fresh cheese, ricotta, and whey samples (Table 2). The methane yield per kg of dry matter consumed by the cows was overestimated in the cream (+5.7%), but not the methane intensity per kg of corrected milk. Both EME traits were slightly overestimated in the scotta (+3.2% for CH_4_/DMI, +4.0% for CH_4_/CM), despite its very low fat content (<0.1%). Lastly, the methane yields of 6- and 12-month ripened cheeses were overestimated by +4.1% and +4.5%, and methane intensity by +5.8% and +5.4%, respectively.

**Figure 1:**
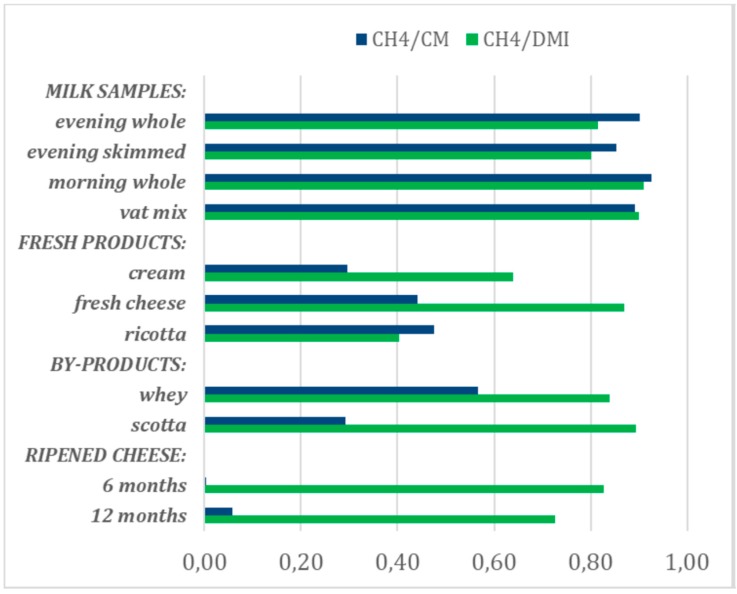
Pearson correlations between the bovine methane intensity per unit fat and protein corrected milk (CH_4_/CM) and methane yield per unit dry matter intake, (CH_4_/DMI) predicted on the reference bovine milk (average of evening and morning whole milk) and those predicted using other milk samples and dairy products and by-products.

The Pearson correlations between the estimates obtained in the reference milk and in each of the 11 alternative dairy matrices are reported in Figure 1, from which it can be seen that all four types of single milk samples tested yielded estimates of both EME traits that were highly correlated (>0.80) with those obtained from the reference milk. Among the fresh products, only cheese yielded estimates that were highly correlated with the reference milk, and only for CH_4_/DMI. The latter trait also correlated well with samples of by-products (whey and scotta) and ripened cheese, although in this case the degree of correlation decreased slightly with the length of ripening.

In the case of methane intensity per kg of corrected milk, estimates from samples of dairy products and by-products failed to achieve high correlations with estimates from the reference milk and reached values around 0.50 only in the case of fresh cheese, ricotta, and whey.

## 4. Discussion

The reasons for selecting [11] equations rather than equations from other studies with greater determination coefficients were discussed in a previous large survey [12] where we tested these equations on five different dairy systems. Moreover, we could not use or compare results achievable with the equations proposed in a more recent meta-analysis [13] because not all FAs were available or in the range of variation of those used for equation development. It should, however, be borne in mind that these [11] equations were derived from a meta-analysis covering 30 different cows’ diets. Moreover, the estimates of five EME traits made using this method were found to be moderately heritable [14] and predictable through proper calibration of the Fourier-transform infrared spectra of milk [24]. In any case, we have presented here a preliminary study on a specific dairy system and cheese-making technology.

### 4.1. Informative Fatty Acids

It could be seen from Table 2 that the proportion of the 3 unsaturated FAs found in the milk samples of this study are greater than those previously found in our previous large survey [12]. This difference should be attributed to the feeding regime [25,26] as it is based on pasture in this study and on indoor feeding in the case of the survey.

Negative correlations between EME and unsaturated long-chain FAs have been frequently found [5,6,27], and are a result of inhibition of fiber fermentation in the rumen, responsible for most of the rumen acetate and methane production, and of inhibition of de novo synthesis of FAs in the mammary gland of cows [28]. The de novo FAs were not directly included in the equations. However, it should be noted that [29] included oleic acid (18:1*c*9) in a multivariate latent explanatory factor (“de novo FA” factor) of detailed milk FA profiles, together with the even mid-chain SFAs, but with an opposite sign. This FA could, therefore, offer an indirect way to represent the relationship between mammary fat synthesis and EME. It’s worth noting that oleic acid is included only in the equation for estimating methane intensity and that its coefficient is the lowest of all.

Being the FA with the highest coefficient and the only one positive in both predicting equations, iso-palmitic acid (C16:0*iso*) has an important role in EME estimation. Rico [30] also found this FA to be positively correlated with EME. Although not highly correlated with de novo FAs, iso-palmitic acid is positively correlated with other branched-chain FAs, often of microbial origin, and negatively correlated with linoleic acid [31].

The study of partitioning of individual milk FAs during the cheese-making process is not an objective of this trial as it was discussed in a previous study [17]. Anyway, it was observed a differential migration of some FAs in different products and by-products, mainly related to the size of fat globules (larger in cream and smaller in scotta). During the ripening of cheese, the modification of FA profile is mainly due to lipolysis caused by native enzyme activity and microbial fermentation [32]. These changes could then lead to changes in the estimation of EME traits.

### 4.2. Trueness and Precision of Estimated Enteric Methane Emissions

The average estimates of methane yield (16.9 g/kg DMI) and methane intensity (11.5 g/kg CM) we obtained from the reference milk produced at pasture were lower than the estimates obtained, using the same method, from milk produced from cows on indoor diets [12], which was due to the greater unsaturated FAs found in the milk from cows on pasture [25] and to their inhibition of rumen microbial activity. Moreover, the estimated EME traits increased during the pasture season in relation to changes in the quantity and quality of fresh herbage available (unpublished results) and particularly to the decrease of its lipid content.

The use of individual milk samples (both morning or evening milk, before or after creaming, or also vat milk at the beginning of the cheese-making process), as seen in Table 2, yields an unbiased estimation of both methane yield and intensity (high trueness). The same result was obtained using samples of fresh cheese, ricotta, whey and cream (only for methane intensity). On the contrary, the sampling of cream (for methane yield), scotta, and ripened cheese led to the overestimation of EME traits. This overestimation is due to the different recovery of the various milk fatty acids in the cheese [33]. In a previous research on the recovery rates of individual FAs from milk to ripened cheese [23], we found the recoveries of saturated 4:0 and 16:0iso to be slightly lower than average, but we found the opposite for the unsaturated FAs examined here.

Validation of the accuracy of using different milk and dairy products and by-products for predicting EME traits should not be limited to the evaluation of their trueness or potential positive (overestimation) or negative (underestimation) biases, but should also take into account, even where matrices have no biased average value, the random errors that may reduce the correlations between the estimates obtained in the reference milk and in the alternative sources of information, i.e., precision of the prediction. The Pearson correlations carried out in this preliminary study take into account possible errors due to the repeatability and reproducibility of FA analysis (lower than 100%), and particularly the effects of different cheese- and ricotta-makings carried out on different dates. The Pearson correlations between the estimates obtained in the reference milk and in each of the 11 alternative dairy matrices as seen from Figure 1, were very variable according to the source of information and EME trait.

Table 3 summarizes the results showed previously in Table 2 and Figure 1 for accuracy, in terms of trueness and precision, with which the alternative sources of information predicted enteric methane yield and intensity in this very preliminary study, although further research under different diet/environment/milk processing conditions and using larger datasets is needed to confirm our results. It worth noting that no information on these issues is available in the literature we are aware of, but this preliminary study showed that methane yield and intensity can be predicted with good accuracy (trueness and precision) from single milk samples, regardless of whether these are fresh milk from evening or morning milkings, milk partially skimmed after overnight creaming, or a mixture of milk heated and cultured in the vat at the beginning of cheese-making. The fresh products of cheese- and ricotta-makings showed a generally good level of trueness, but low precision in predicting both EME traits, and this could require an increase in the number of samples analyzed to obtain a precision similar to that achieved with reference milk.

The overestimation of methane yield from the fatty acid profile of cream could be accounted for by specific research in which a proper correction factor is formulated on the basis of the different recovery rates in the cream of the informative fatty acids present in milk. A moderate rate of precision, on the other hand, could be accounted for only by increasing the number of samples analyzed. Of the two by-products analyzed, whey may be considered a viable alternative source of information for predicting both EME traits, whereas scotta overestimated both traits and had a low level of precision (also because of its very low fat content). Cheeses ripened for long periods were revealed to be poor sources of information for predicting EME traits, although with specific correction factors, six-month-ripened cheeses could constitute an acceptable source of information for predicting methane emitted per kg of dry matter consumed by lactating cows.

## 5. Conclusions

Despite the need for further research on different animal, environment, and cheese-making conditions, it could be concluded that this preliminary study outlines the possibilities offered by the fatty acid profile of milk and dairy products and byproducts for easily monitoring their impact on global climate change along the dairy chain. Different types of single milk samples (taken from morning or evening milking, made of whole or creamed milk, sampled fresh or after mixing, heating and culturing for cheese-making) have proven to yield comparable results as the reference whole fresh milk sample obtained mixing milk from morning and evening milking. This allows for a comparison of the carbon footprint of different milk suppliers. Moreover, different dairy products (cream, ricotta, fresh and ripened cheeses) and byproducts (whey) can also be used, increasing the number of samples or using some correction factor, for comparing different milk processors or dairy retailers along the dairy chain.

## Figures and Tables

**Table 1:** Descriptive statistics of the bovine milk composition and of the dairy produce yield.

Trait	Mean	SD
Milk composition:		
Fat, %	3.57	0.84
Protein, %	3.70	0.09
Casein, %	2.74	0.11
Lactose, %	4.87	0.13
SCS ^1^, U/mL	3.59	0.54
pH	6.52	0.03
Product yield traits (% on processed milk):		
Cream	6.32	1.56
Fresh Cheese	14.22	0.78
Ricotta	4.97	0.72
Ripened cheese (6 months)	9.60	0.53
Ripened cheese (12 months)	9.14	0.60

^1^ SCS = log_2_(SCC/100,000) + 3.

**Table 2:** Content of informative fatty acids and derived estimated methane yield (per unit dry matter intake, DMI) and intensity (per unit corrected milk, CM) of reference bovine milk (average of whole evening and morning samples) and of other milk samples and dairy products and by-products.

Dairy Product and By-Product	Informative Fatty Acids ^1^ (g/100g FA):					Predicted Methane Emissions (g/kg):	
	C4:0	C16:0*iso*	C18:1*t*10-11	C18:1*c*9	C18:2*c*9,*c*12	CH_4_/DMI	CH_4_/CM
Reference bovine milk	2.92	0.29	4.52	24.41	2.54	16.94	11.49
Single milk samples ^2^							
Evening whole	2.89	0.29	4.63	25.17 **	2.58	16.77	11.33
Evening skimmed	2.97	0.29	4.59	25.39 ***	2.59	16.81	11.21
Morning whole	2.96	0.28	4.41	23.65 **	2.49	17.11	11.66
Vat mix	3.10	0.30	4.49	24.12	2.59	17.01	11.44
Fresh products ^2^							
Cream	3.22 *	0.30 **	4.09 ***	23.02 ***	2.35	17.91 ***	11.80
Fresh cheese	3.25 *	0.29	4.39	23.80 *	2.57	17.02	11.25
Ricotta	3.23 *	0.29	4.48	23.87 *	2.45	17.21	11.27
By-products ^2^							
Whey	3.13	0.29	4.44	24.14	2.45	17.25	11.39
Scotta ^3^	3.61 ***	0.30 *	3.18 ***	22.30 ***	3.13	17.48 *	11.95 *
Ripened cheese ^2^							
6 months	2.77	0.29	4.29 **	23.18 ***	2.31	17.63 ***	12.16 **
12 months	2.79	0.29	4.13 ***	23.53 ***	2.35	17.70 ***	12.11 **
Cheese-making effect ^4^	***	***	***	***	***	***	***
RMSE	0.27	0.02	0.19	0.53	0.13	0.18	0.33

^1^ Informative milk FAs are the fatty acids included as independent variables in the equations used to estimate the enteric methane emissions (van Lingen et al., 2014); ^2^ The asterisks: *: *p* < 0.05; **: *p* < 0.01; ***: *p* < 0.001 refer to the significance of the contrast between the reference milk and the alternative information source (single milk samples, dairy products and by-products); ^3^ Scotta = residual liquid from Ricotta production; ^4^ The asterisks refer to the differences observed from the first to the last (7th) cheese- and ricotta-making sessions controlled every two weeks during summer transhumance.

**Table 3:** Accuracy of the predictions of bovine enteric methane yield (CH_4_/DMI) and enteric methane intensity per kg corrected milk (CH_4_/CM) obtained from the fatty acid profile of alternative sources of information (single milk samplings, and dairy products and by-products) respect to the reference value obtained from fatty acid profile of the average of samplings of unprocessed bovine milk from morning and evening milkings. Accuracy is evaluated in terms of trueness (bias) and precision (error).

Dairy Products and By-Products	CH_4_/DMI		CH_4_/CM	
	Trueness ^1^	Precision ^2^	Trueness ^1^	Precision ^2^
Single milk samples:				
Evening whole	Good	High	Good	High
Evening skimmed	Good	High	Good	High
Morning whole	Good	High	Good	High
Vat mix	Good	High	Good	High
Fresh products:				
Cream	Overestimate 6%	Moderate	Good	Low
Fresh cheese	Good	High	Good	Low
Ricotta	Good	Low	Good	Low
By-products:				
Whey	Good	High	Good	Moderate
Scotta ^3^	Overestimate 3%	High	Overestimate 4%	Low
Ripened cheese:				
6 months	Overestimate 4%	High	Overestimate 6%	Very low
12 months	Overestimate 4%	Moderate	Overestimate 5%	Very low

^1^ Measures the difference (bias) between the least square mean (LSM) of the enteric methane emissions (EME) trait obtained from an alternative source of information (single milk sample, dairy product or by-product) and that obtained from the reference milk (Table 2): “Good” means that the difference is not significant (*p* > 0.05); “Overestimate” means that the difference between the two LSM is significantly greater (*p* < 0.05) in the alternative than in the reference milk; ^2^ Measures the correlation (error) between the individual estimates obtained from an alternative source of information (single milk sample, dairy product or by-product) and those obtained from the reference milk (Figure 1): “High”, the correlation is >0.80; “Moderate” means that the correlation is 0.50 to 0.80; “Low” means that the correlation is 0.20 to 0.50; “Very low” means that the correlation is <0.20; ^3^ Scotta = residual liquid from Ricotta production.
